# Impact of intravascular hemolysis on functional and molecular alterations in the urinary bladder: implications for an overactive bladder in sickle cell disease

**DOI:** 10.3389/fphys.2024.1369120

**Published:** 2024-07-19

**Authors:** Tammyris Helena Rebecchi e Silveira, Dalila Andrade Pereira, Danillo Andrade Pereira, Fabiano Beraldi Calmasini, Arthur L. Burnett, Fernando Ferreira Costa, Fábio Henrique Silva

**Affiliations:** ^1^ Laboratory of Pharmacology, São Francisco University Medical School, Bragança Paulista, Brazil; ^2^ Department of Pharmacology, Escola Paulista de Medicina, Universidade Federal de São Paulo, São Paulo, Brazil; ^3^ The James Buchanan Brady Urological Institute and Department of Urology, The Johns Hopkins School of Medicine, Baltimore, MD, United States; ^4^ Hematology and Hemotherapy Center, University of Campinas, Campinas, Brazil

**Keywords:** cyclic guanosine monophosphate, nitric oxide, oxidative stress, NADPH oxidase, urinary dysfunction

## Abstract

Patients with sickle cell disease (SCD) display an overactive bladder (OAB). Intravascular hemolysis in SCD is associated with various severe SCD complications. However, no experimental studies have evaluated the effect of intravascular hemolysis on bladder function. This study aimed to assess the effects of intravascular hemolysis on the micturition process and the contractile mechanisms of the detrusor smooth muscle (DSM) in a mouse model with phenylhydrazine (PHZ)-induced hemolysis; furthermore, it aimed to investigate the role of intravascular hemolysis in the dysfunction of nitric oxide (NO) signaling and in increasing oxidative stress in the bladder. Mice underwent a void spot assay, and DSM contractions were evaluated in organ baths. The PHZ group exhibited increased urinary frequency and increased void volumes. DSM contractile responses to carbachol, KCl, α-β-methylene-ATP, and EFS were increased in the PHZ group. Protein expression of phosphorylated endothelial NO synthase (eNOS) (Ser-1177), phosphorylated neuronal NO synthase (nNOS) (Ser-1417), and phosphorylated vasodilator-stimulated phosphoprotein (VASP) (Ser-239) decreased in the bladder of the PHZ group. Protein expression of oxidative stress markers, NOX-2, 3-NT, and 4-HNE, increased in the bladder of the PHZ group. Our study shows that intravascular hemolysis promotes voiding dysfunction correlated with alterations in the NO signaling pathway in the bladder, as evidenced by reduced levels of p-eNOS (Ser-1177), nNOS (Ser-1417), and p-VASP (Ser-239). The study also showed that intravascular hemolysis increases oxidative stress in the bladder. Our study indicates that intravascular hemolysis promotes an OAB phenotype similar to those observed in patients and mice with SCD.

## 1 Introduction

Sickle cell disease (SCD), an autosomal recessive genetic disorder, is characterized by abnormal hemoglobin S (HbS) production due to a single amino acid substitution in the β-globin chain (Kato et al., 2018). This genetic mutation triggers the polymerization of HbS under hypoxic or dehydrated conditions, forming sickle-shaped erythrocytes. These altered cells exhibit increased stiffness and a reduced lifespan, leading to intravascular and extravascular hemolysis, which are critical features of SCD and contribute to its diverse clinical manifestations (Kato et al., 2018). A significant molecular consequence of intravascular hemolysis is the reduction of nitric oxide (NO) bioavailability due to direct NO-hemoglobin interaction and increased reactive oxygen species (ROS) production, which act as NO scavengers (Reiter et al., 2002; Vona et al., 2021). This reduction in NO is associated with various severe SCD complications, including leg ulceration, pulmonary hypertension, priapism, and overactive bladder (OAB) (Nolan et al., 2005; Kato et al., 2006; Cita et al., 2016).

The functions of the urinary bladder, encompassing urine storage and voiding, are regulated by a complex interplay of neurotransmitters (Andersson and Arner, 2004). OAB, a clinical condition marked by persistent urgency to urinate, may occur with or without urge incontinence and is commonly accompanied by increased urination frequency and nocturia (Eapen and Radomski, 2016). Notably, in SCD patients, the prevalence of OAB is significant, with clinical studies suggesting that up to 40% of these patients may exhibit symptoms of OAB (Portocarrero et al., 2012; Anele et al., 2015). A common contributor to OAB is the heightened contraction of the detrusor smooth muscle during the urine storage phase, leading to detrusor overactivity (Michel and Chapple, 2009).

The NO-cyclic guanosine monophosphate (cGMP) signaling pathway plays an essential role in the normal functioning of the urinary tract. NO, synthesized by endothelial NO synthase (eNOS) and neuronal NO synthase (nNOS), is crucial for maintaining the tone and functionality of detrusor smooth muscle (Burnett et al., 1997; Mónica et al., 2008; Karakus et al., 2019). NO deficiency has been linked with the OAB phenotype and increased detrusor smooth muscle contraction in SCD mice and various experimental models (Khan et al., 1999; Mónica et al., 2011; Leiria et al., 2013; Leiria et al., 2014; Karakus et al., 2019; Musicki et al., 2019; Karakus et al., 2020; Lee et al., 2021). Furthermore, increased superoxide production by the NOX-2 isoform of NADPH oxidase, which acts by activating NO, also contributes to the pathophysiology of OAB in animal models (Alexandre et al., 2016; 2018; Akakpo et al., 2017; de Oliveira et al., 2022) but has not yet been evaluated in the lower urinary tract in SCD.

To date, previous studies have used SCD transgenic mice to investigate bladder alterations (Claudino et al., 2015; Karakus et al., 2019; 2020; Musicki et al., 2019). These studies reported voiding dysfunction and detrusor hypercontractility associated with reduced NO bioavailability in the bladder. However, the exclusive effects of intravascular hemolysis on the bladder have not been independently analyzed. Given the critical role of intravascular hemolysis in SCD and its potential impact on the NO signaling pathway in the bladder, we hypothesize that intravascular hemolysis contributes significantly to micturition dysfunction.

The phenylhydrazine (PHZ)-induced hemolysis model permits precise control over the onset and intensity of hemolysis (Lim et al., 1998; Dutra et al., 2014; Gotardo et al., 2023), enabling a direct correlation between hemolysis and the observed functional and molecular changes in the bladder. This model is precious when the aim is to study the exclusive effect of intravascular hemolysis, as SCD mice exhibit additional alterations beyond intravascular hemolysis. This study endeavors to fill this critical gap in knowledge, providing an in-depth understanding of the mechanisms underlying intravascular hemolysis-induced micturition dysfunction.

This study is designed to delineate the consequences of intravascular hemolysis on the micturition process and the contractile mechanisms of the detrusor smooth muscle in a mouse model of PHZ-induced hemolysis. Furthermore, we investigate the role of intravascular hemolysis in elevating ROS production in the bladder, as well as to assess changes in phosphorylated eNOS (Ser-1177), phosphorylated nNOS (Ser-1417), and phosphorylated vasodilator-stimulated phosphoprotein (p-VASP Ser-239).

## 2 Materials and methods

### 2.1 Ethical approval

All animal study protocols in this study were approved by the Ethics Committee on Animal Use of the University of San Francisco (CEUA/USF, Permit number V3:008.06.2021).

### 2.2 Animals and treatment

Animal procedures and experimental protocols were performed in accordance with the ethical principles in animal research adopted by the Brazilian College for Animal Experimentation and followed the Guide for the Care and Use of Laboratory Animals. All mouse strains were originally purchased from Jackson Laboratories (Bar Harbor, ME). Characterization and breeding were performed at the Multidisciplinary Center for the Investigation of Biological Science in Laboratory Animals of the University of Campinas. We used C57BL/6 male mice (control), aged 3–4 months old, housed five per cage on a 12 h light–dark cycle.

We injected PHZ at 50 mg/kg in C57BL/6 mice intraperitoneally to induce intravascular hemolysis. The mice were reinjected with 50 mg/kg 8 h later and were sacrificed 4 days after starting PHZ treatment. Control mice were treated with the saline vehicle simultaneously with the PHZ group (Henrique Silva et al., 2018).

### 2.3 Void spot assay

Mice were moved individually to empty mouse cages with precut qualitative filter paper (250 g) on the bottom. They were provided with food but no water. After 4 hours, the filter papers were removed and allowed to dry before being imaged using UV light transillumination and captured using the ChemiDoc MP imaging system with Image Laboratory software (Bio-Rad Laboratories, Hercules, CA). Captured images were saved in grayscale-tagged image file format (TIFF) (Figure 2A) and analyzed using ImageJ Software (National Institute of Health, Bethesda-MD, United States of America). ImageJ particle analysis was performed on >0.02 cm^2^ spots to reduce areas of non-specific fluorescence and artifacts that may have been created by debris and feces (Keil et al., 2016). A linear standard measurement curve was used to convert void spot areas to volumes, and total volumes were normalized to body weight. Assays were performed for each animal between 9 a.m. and 2 p.m.

### 2.4 Functional studies of bladder strips and concentration–response curves

Two longitudinal detrusor smooth muscle strips with intact urothelium were obtained from each bladder. The strips were mounted in 5 mL myograph organ baths (Danish Myo Technology, Aarhus, Denmark) containing Krebs–Henseleit solution composed of 117 mM NaCl, 4.7 mM KCl, 2.5 mM CaCl_2_, 1.2 mM MgSO_4_, 1.2 mM KH_2_PO_4_, 25 mM NaHCO_3_, and 11 mM glucose, continuously bubbled with a mixture of 95% O_2_ and 5% CO_2_ (pH 7.4) at 37°C. Changes in isometric force were recorded using a PowerLab Data Acquisition System (Software LabChart, version 7.0, ADInstruments, MA, United States of America).The resting tension was adjusted to 5 mN at the beginning of the experiments. The equilibration period was 60 min, and the bathing medium was changed every 15 min.

Cumulative concentration-response curves for the full muscarinic agonist carbachol (1 nM–30 µM) and potassium chloride (KCl; 1–300 mM) were obtained in detrusor strips. In separate experiments, electrical field stimulation (EFS)-induced contraction (20 V, 10 s of stimulation at varying frequencies, a 2-min interval between each pulse) was carried out. Non-cumulative concentration–response curves were also made for the purinergic agonist (P2X), α-β-methylene-ATP (1 μM, 3 μM, and 10 µM).

Non-linear regression analysis used GraphPad Prism (GraphPad Software, San Diego, CA, United States of America). Maximal response (E_max_) data were normalized to the wet weight of the respective urinary bladder strips. Using GraphPad Prism software, EC_50_ values, represented as the negative logarithm (pEC50), were calculated by fitting the concentration–response relationship to a sigmoidal model (log-concentrations vs. response).

### 2.5 Western blot analysis

The separation of proteins from biological samples of tissue homogenates (detrusor) was performed through electrophoresis in 4%–20% polyacrylamide with 0.1% sodium sulfate (SDS-Page). Then, the protein bands were transferred electrophoretically into a submerged nitrocellulose membrane system. Non-specific protein binding to nitrocellulose was reduced by “overnight” pre-incubation of the membrane with a blocking solution (5% milk powder, 10 mm Tris, 100 mm NaCl, and 0.02% Tween 20). The bladder from each mouse was homogenized in lysis buffer and centrifuged at 12,000 g for 20 min at 4°C. Homogenates containing 70 μg of total proteins were run on 4%–20% Tris-HCl gels (Bio-Rad Laboratories, Hercules, CA, United States of America) and transferred to a nitrocellulose membrane. Non-fat dry milk (5%) (Bio-Rad) in Tris-buffered saline/Tween was used for 60 min at 24°C to block non-specific binding sites. Membranes were incubated for 15–18 h at 4°C with the following antibodies: monoclonal anti-3-nitrotyrosine (3-NT; 1:3000, Abcam), polyclonal anti-4-HNE antibody (1:3000, Abcam), anti-NOX-2 antibody (1:1000, Sigma-Aldrich), monoclonal anti-phospho(p)-eNOS (Ser-1177) antibody (1:1000, Cell Signaling), polyclonal anti-eNOS antibody (1:1000, Cell Signaling), polyclonal anti-phospho(p)-VASP (Ser-239) (1:1000, Cell Signaling), monoclonal anti-VASP antibody (1:1000, Cell Signaling), polyclonal phospho(p)-nNOS antibody (Ser-1417) (1:1000, Abcam), nNOS (1:1000, Millipore), and β-actin (1:5000, Santa Cruz Biotechnology). Densitometry was analyzed using ImageJ software (National Institute of Health, Bethesda-MD, United States of America). Quantified densitometry results were normalized to β-actin.

### 2.6 Drugs

Carbachol, α-β-methylene-ATP, PHZ, and KCl were purchased from Sigma-Aldrich (St Louis, MO, United States of America. All reagents were required to be of analytical grade. Deionized water was used as a solvent, and working solutions were diluted prior to use.

### 2.7 Statistical analysis

The GraphPad Prism Program (GraphPad Software Inc.) was used for statistical analysis. Data are expressed as the mean ± SEM of N experiments. Statistical comparisons were made using the Student’s unpaired *t*-test. A value of *p* < 0.05 was considered statistically significant.

## 3 Results

### 3.1 Hematological parameters

**FIGURE 1:**
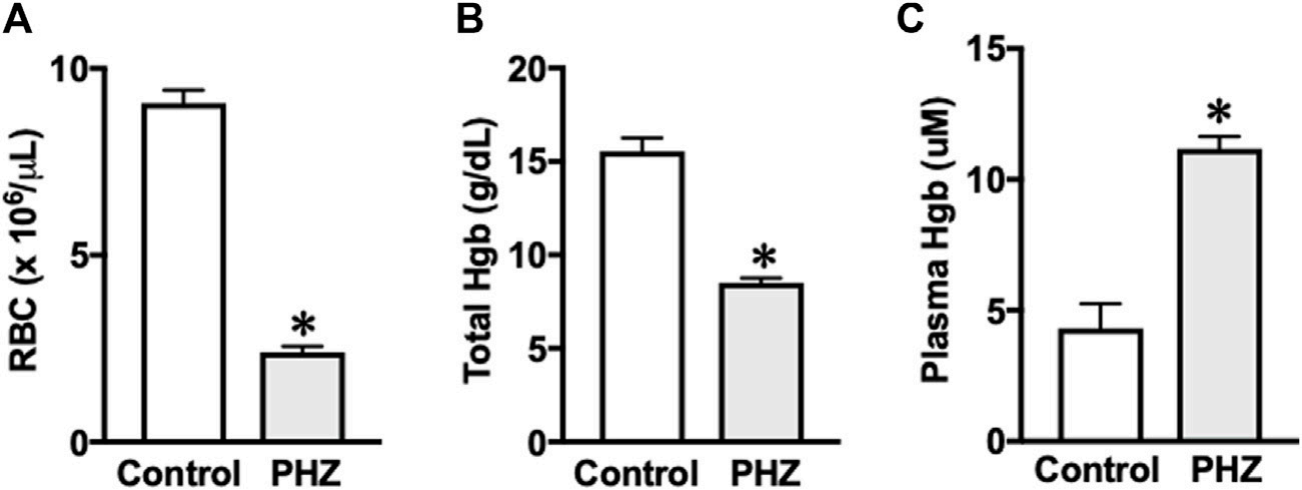
**(A)** Red blood cell, **(B)** hemoglobin, and **(C)** plasma hemoglobin. Data are shown as the mean ± SEM of 5–7 mice per group. *p < 0.05 vs. control group.

Mice treated with PHZ exhibited significantly reduced levels of red blood cells (Figure 1A) and total hemoglobin (Figure 1B) compared to the control group (*p* < 0.05). Furthermore, there was a marked increase in plasma hemoglobin concentrations in the PHZ group (*p* < 0.05) compared to the control (Figure 1C), confirming the occurrence of intravascular hemolysis.

### 3.2 Intravascular hemolysis leads to increased urinary frequency and increased void volumes

**FIGURE 2:**
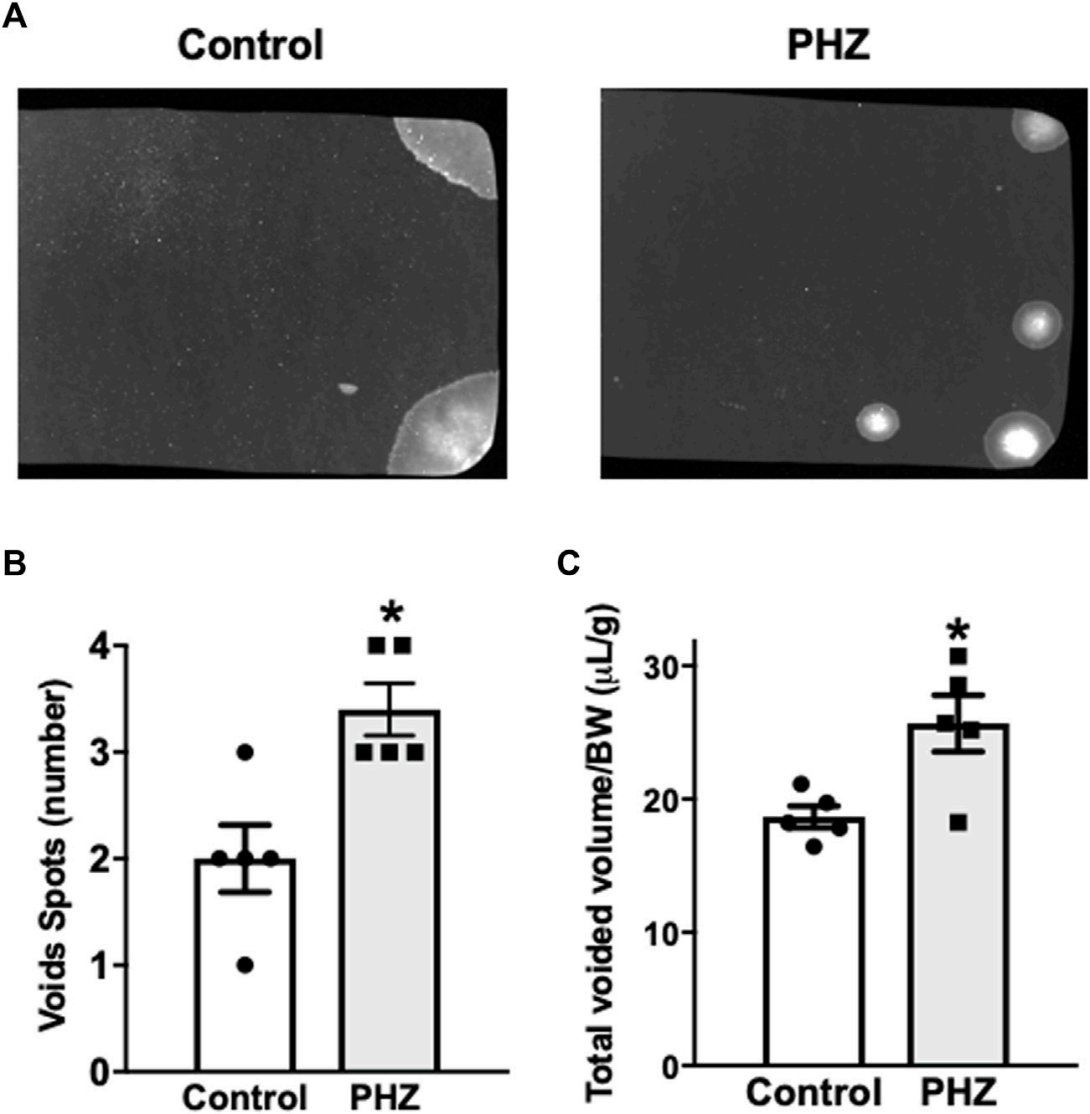
**(A)** Representative void spot assays, **(B)** number of spots, and **(C)** normalized voided volume in control and PHZ mice. Data are shown as the mean ± SEM of five mice per group. *p < 0.05 vs. control group.

Figure 2A presents filter paper examples from the control and PHZ-treated mice. The PHZ group showed a significant increase in urinary spots compared to the control group (*p* < 0.05), indicating a hyperactive voiding behavior (Figure 2B). Additionally, the total void volumes produced by the PHZ-treated mice were significantly greater than those of the control mice (*p* < 0.05) (Figure 2C).

### 3.3 Intravascular hemolysis leads to detrusor hypercontractility

**FIGURE 3:**
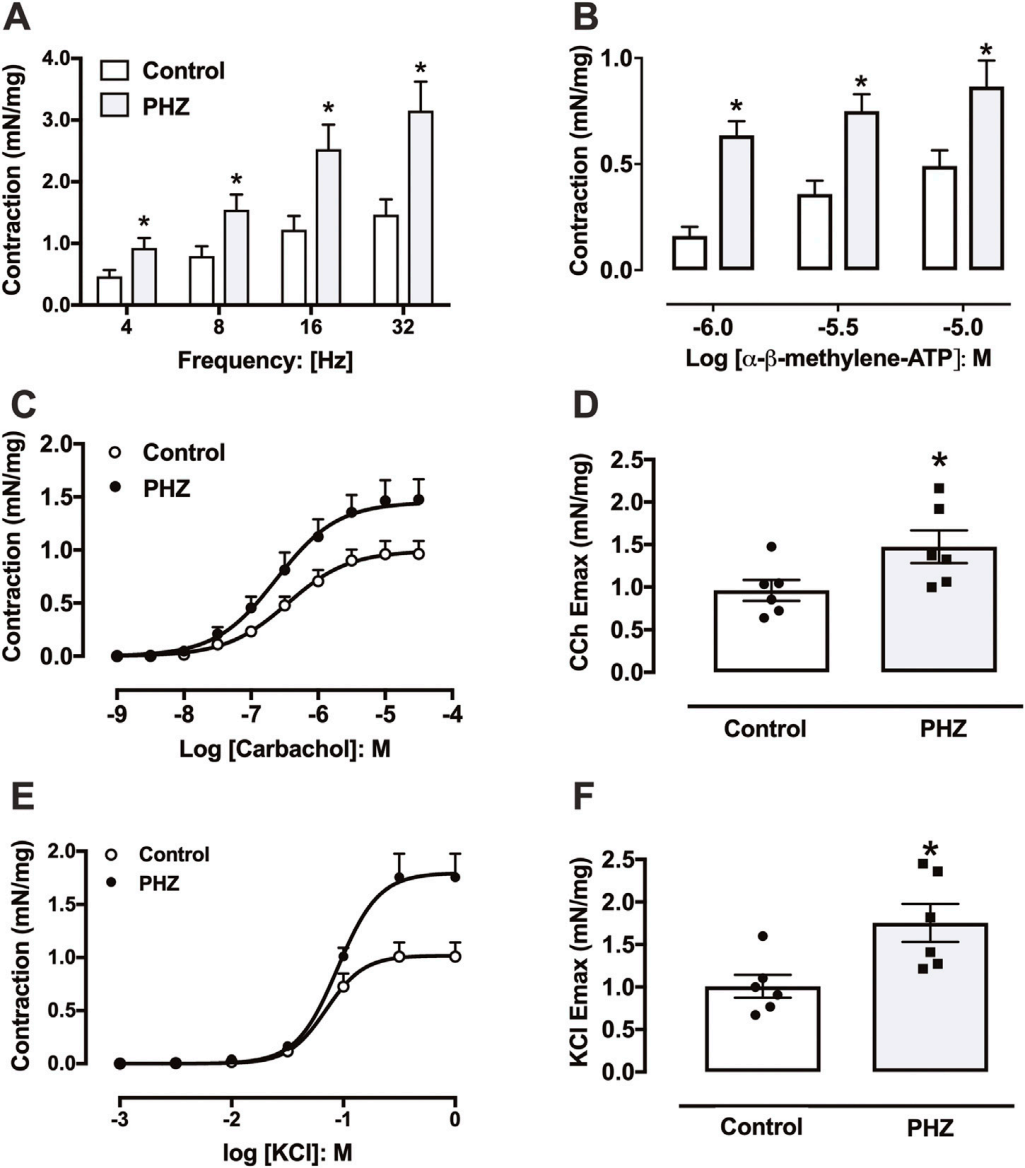
Contractile responses to **(A)** electrical field stimulation, **(B)** α-β-methylene-ATP, **(C)** carbachol, and **(E)** KCl in the bladder from control and PHZ mice. **(D)** E_max_ values for **(D)** carbachol and **(F)** KCl. Data are shown as the mean ± SEM of six mice per group. *p < 0.05 vs. control group.

EFS of 4–32 Hz induced frequency-dependent contractions in the detrusor smooth muscle in both control and PHZ-treated mice. Notably, the PHZ group exhibited significantly higher contractions at all frequencies compared to the control group (*p* < 0.05) (Figure 3A).

Contractile response to α-β-methylene-ATP in the detrusor smooth muscle was assessed through non-cumulative concentration-effect curves (1 μM, 3 μM, and 10 μM) for both groups (Figure 3B). Detrusor smooth muscle from PHZ-treated mice displayed a significantly enhanced contractile response to α-β-methylene-ATP at all tested concentrations compared to the control (*p* < 0.05) (Figure 3B).

Contraction responses to carbachol were evaluated in detrusor smooth muscle from both PHZ and control mice through concentration-effect curves for the agonist (1 nM–30 μM) (Figure 3C). The maximal contractile response (E_max_) elicited by carbachol was significantly greater in the detrusor smooth muscle of the PHZ group (*p* < 0.05) than that of the control group (Figure 3D), with no notable differences in potency (pEC50) between the control (6.45 ± 0.12) and PHZ-treated mice (6.62 ± 0.09). Similarly, KCl induced concentration-dependent contractions in both groups (Figure 3E). Notably, E_max_ to KCl was significantly greater in the PHZ group than in the control group (Figure 3F). No significant differences in potency (pEC50) for KCl were observed between the control group (1.01 ± 0.06) and the PHZ-treated group (6.62 ± 0.09).

**FIGURE 4:**
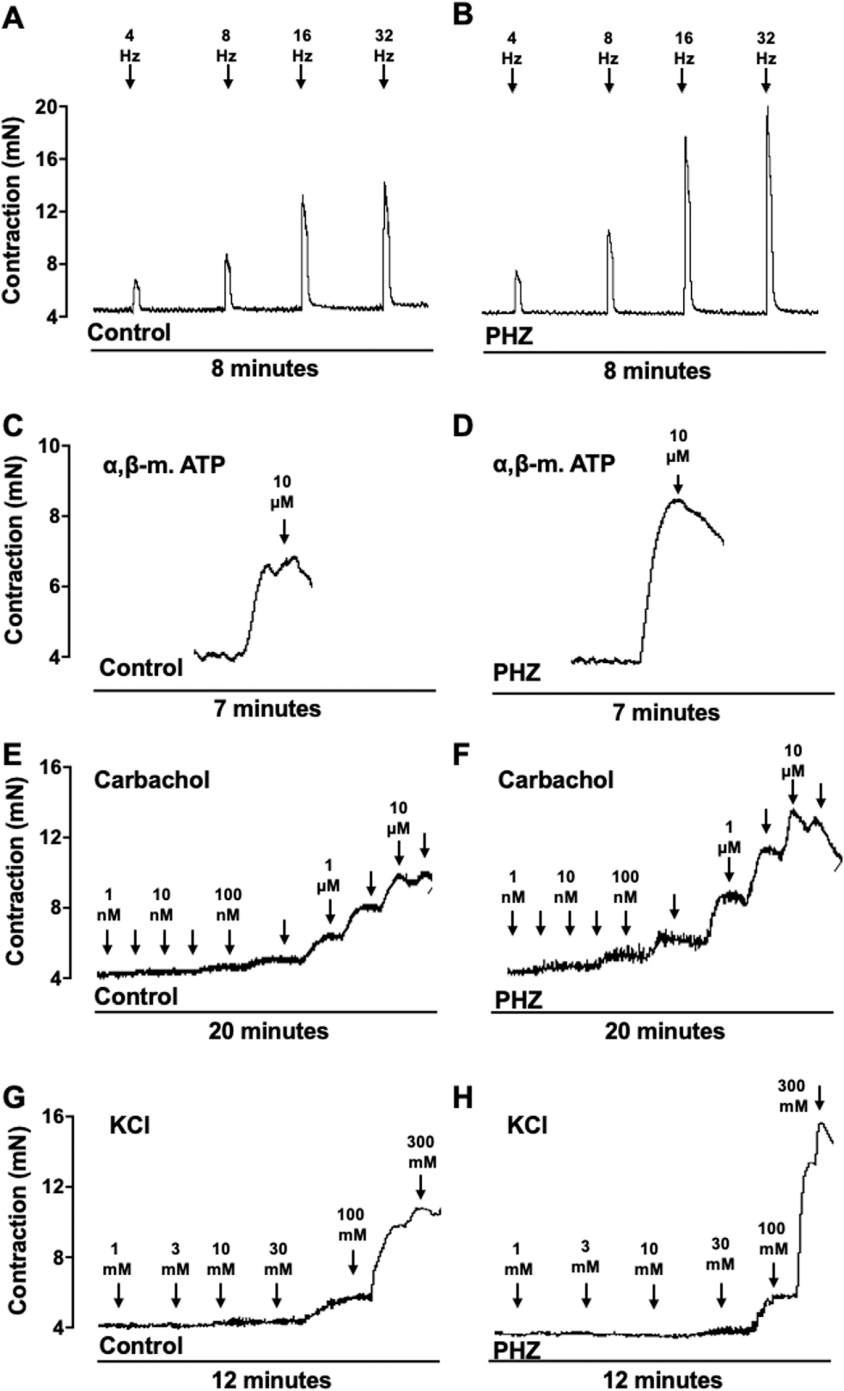
Representative tracings of contraction response to EFS, α-β-methylene-ATP, carbachol, and KCl from control and PHZ mice.

Representative traces of responses to EFS, α-β-methylene-ATP, carbachol, and KCl are shown in Figure 4.

### 3.4 Intravascular hemolysis decreased protein expressions of p-eNOS (Ser-1177), p-nNOS (Ser-1417), and p-VASP (Ser-239) in the mouse bladder

**FIGURE 5:**
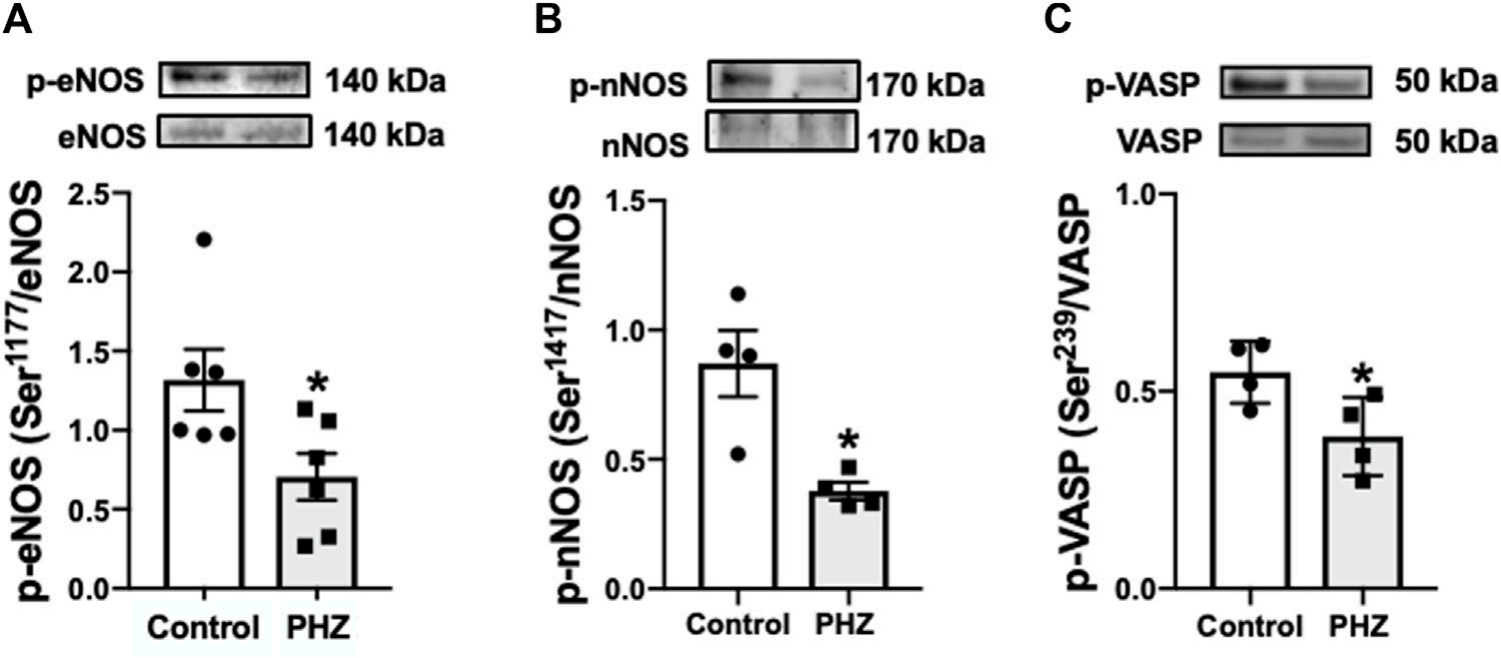
Representative images of Western blotting (top panels) and protein values (bottom panels) for **(A)** p-eNOS (ser-1177) (n = 6), **(B)** p-nNOS (ser-1417) (n = 4), and **(C)** p-VASP (Ser-239) (n = 4) in homogenates of bladder from control and PHZ mice. Data are shown as the mean ± SEM. *p < 0.05 vs. control group.

Protein expression of activated (phosphorylated) forms of eNOS (p-eNOS Ser-1177), nNOS (p-nNOS Ser-1417), and VASP (p-VASP Ser-239) was investigated to understand the impact of intravascular hemolysis on signaling of nitric oxide (NO) in the bladder. These enzymes play fundamental roles in the regulation of smooth muscle tone: eNOS and nNOS are responsible for the production of NO, an important mediator of smooth muscle relaxation, while VASP is a substrate of the cGMP-protein kinase G (PKG) pathway, reflecting the activity of NO-cGMP-PKG signaling. PKG, activated by cGMP, is essential for mediating the effects of NO on smooth muscle, promoting relaxation, and directly influencing bladder function (Oelze et al., 2000; Francis et al., 2010). In the PHZ-treated mice, the activated (phosphorylated) forms of p-eNOS (Ser-1177), p-nNOS (Ser-1417), and p-VASP (Ser-239) were significantly reduced in the bladder compared to the control group (*p* < 0.05), as shown in Figure 5. These results suggest that intravascular hemolysis compromises NO signaling in the bladder, potentially contributing to voiding dysfunction.

### 3.5 Intravascular hemolysis leads to increased oxidative stress markers in the mouse bladder

**FIGURE 6:**
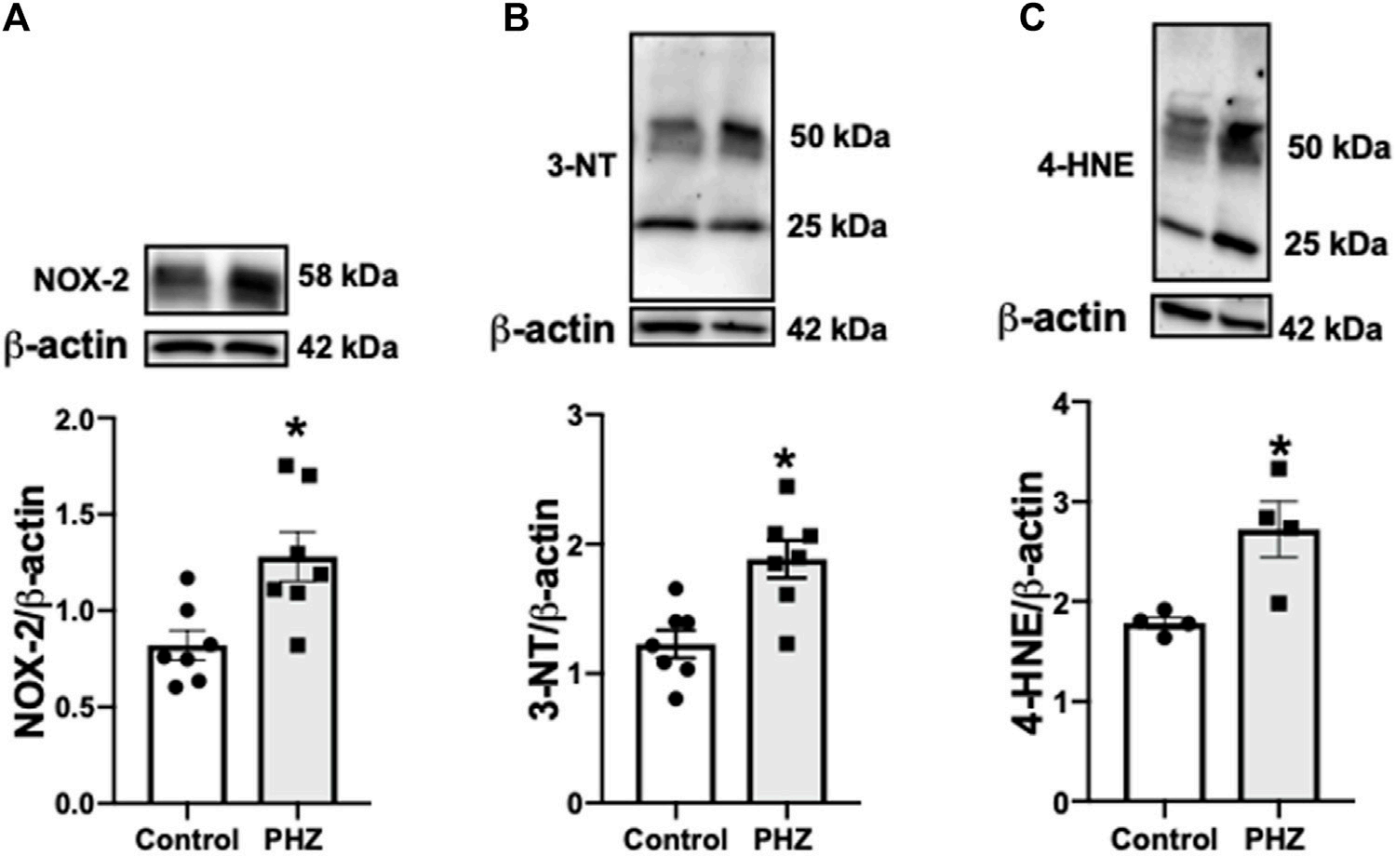
Representative images of Western blotting (top panels) and protein values (bottom panels) of **(A)** NOX-2 (n = 7), **(B)** 3-NT (n = 7), and **(C)** 4-HNE (n = 4) in homogenates of the bladder from control and PHZ mice. Data are shown as the mean ± SEM. *p < 0.05 vs. control group.

To assess the impact of intravascular hemolysis on oxidative stress in the bladder, the protein expression of oxidative stress markers, including NOX-2, 3-NT, and 4-HNE, was examined. NOX-2 is an important enzyme in ROS production, while 3-NT and 4-HNE are products of oxidative damage to proteins, serving as markers of nitrosative and oxidative stress, respectively (Pacher et al., 2007; Vermot et al., 2021). In the PHZ-treated mice, there was a significant increase in the protein expression of oxidative stress markers NOX-2, 3-NT, and 4-HNE in the bladder compared to the control group (*p* < 0.05), as shown in Figure 6. This increase in oxidative stress markers indicates that intravascular hemolysis promotes a pro-oxidative environment in the bladder, which may impair organ function and contribute to the development of OAB phenotypes such as those observed in SCD models.

## 4 Discussion

In this study, PHZ-induced hemolysis in mice led to significant hematological changes, mirroring aspects of SCD. The increased urinary frequency and volume increase in PHZ-treated mice aligns with OAB symptoms, suggesting a link between intravascular hemolysis and bladder dysfunction. Notably, the enhanced contractility of the detrusor muscle in these mice indicates a direct impact of hemolysis on bladder smooth muscle activity. The decreased expression of p-eNOS (Ser-1177), nNOS (Ser-1417), and p-VASP (Ser-239) in the bladder tissue indicates a dysregulated NO signaling pathway in the impaired bladder function. Additionally, elevated oxidative stress markers in the bladders of PHZ-treated mice reinforce the role of oxidative stress in OAB phenotypes.

A central aspect of SCD is intravascular hemolysis, where red blood cell contents like hemoglobin, arginase, and other cellular components are released into the plasma (Kato et al., 2017). The PHZ-induced intravascular hemolysis model in mice is widely utilized for assessing the specific effects of intravascular hemolysis (Vannucchi et al., 2001; Henrique Silva et al., 2018; Iacopucci et al., 2022; Gotardo et al., 2023). Our study corroborates previous findings and confirms that PHZ-induced intravascular hemolysis in mice led to significant hematological changes, closely replicating the hemolytic environment seen in SCD. Free hemoglobin (HbFe^2+^) in the plasma or interstitial space quickly reacts with NO, leading to nitrate production and the formation of methemoglobin (HbFe^3+^), the oxidized form of hemoglobin (Reiter et al., 2002). This process significantly reduces NO bioavailability, contributing to tissue damage (Gladwin et al., 2012; Kato et al., 2017; Kato et al., 2018). An efficient pharmacological strategy that has been studied to limit the effects of hemoglobin involves treatment with haptoglobin. This plasma protein binds to free hemoglobin, forming a complex that is then cleared from circulation by the macrophages of the reticuloendothelial system (Buehler et al., 2020).

NO plays a crucial role in the physiology of the lower urinary tract, with its diminished bioavailability linked to micturition dysfunction. The OAB in SCD mice is associated with decreased expression of phosphorylated eNOS at its positive regulatory site Ser-1177 and phosphorylated nNOS at its positive regulatory site Ser-1412 in the bladder. Similarly, in our study, PHZ mice displayed decreased expression of p-eNOS (Ser-1177) and p-nNOS (Ser-1417), indicating lower NO production in the bladder. NO activates soluble guanylate cyclase (sGC) in smooth muscle, enhancing cGMP production. cGMP activates protein kinase G, which phosphorylates VASP at Ser-239, a reliable biomarker for monitoring the NO-stimulated cGMP-protein kinase G pathway (Oelze et al., 2000; Francis et al., 2010). In our study, protein expression for p-VASP (Ser-239) was lower in the bladder in the PHZ group, indicating decreased cGMP levels. Mice lacking nNOS exhibit bladder hypertrophy, dysfunctional urinary outlets, and increased urinary frequency (Burnett et al., 1997). Additionally, rats treated chronically with a NOS inhibitor develop an OAB phenotype (Mónica et al., 2008; Mónica et al., 2011). Altered micturition patterns have been previously reported in cGMP-dependent protein kinase I gene-deficient mice (Persson et al., 2000). PHZ-treated mice exhibited increased urinary spots and higher total void volumes. These results align with findings from animal models lacking both eNOS and nNOS, as well as SCD mice (Karakus et al., 2019; Musicki et al., 2019; Karakus et al., 2020), reinforcing the importance of NO pathways in urinary function. A prior study speculated that the augmented urine volumes observed in double-NOS (eNOS and nNOS) or triple-NOS (eNOS, nNOS, and iNOS) knockout mice could be attributed to impairments in renal function, specifically in the ability to concentrate urine, leading to polyuria (Morishita et al., 2005).

Acetylcholine, primarily through muscarinic M3 receptors, is the primary excitatory neurotransmitter in the parasympathetic nerve endings of detrusor smooth muscle (Andersson and Arner, 2004). Co-stored and co-released ATP with acetylcholine also play a significant role in nerve-mediated bladder contraction, contributing to efficient urine elimination (Burnstock, 2011). In our study, detrusor contractions induced by EFS were significantly higher in the PHZ-treated group. In parallel, the responses of detrusor smooth muscle to both muscarinic and purinergic receptor agonists (carbachol and α, β-methylene-ATP, respectively), as well as to the receptor-independent agent KCl, were also increased in PHZ-treated mice. These findings indicate that intravascular hemolysis leads to detrusor hypercontractility. The increase in detrusor muscle contraction is likely secondary to the low accumulation of cGMP in bladder tissue, a well-known secondary messenger that counteracts the contractile mechanisms of smooth muscle (Mónica and Antunes, 2018). Rats treated chronically with a non-selective inhibitor of NOS (L-NAME) show increased detrusor contraction induced by muscarinic receptor agonists (Mónica et al., 2008), as well as animal models deficient in both eNOS and nNOS (Karakus et al., 2019), highlighting the importance of the integrity of the NO pathway in bladder function.

Increased oxidative stress, characterized by elevated ROS production or reduced antioxidant capacity, is associated with the development of OAB in experimental models and participates in the pathophysiology of SCD (Alexandre et al., 2016; Silva et al., 2016; Akakpo et al., 2017; Alexandre et al., 2018; Vona et al., 2021; de Oliveira et al., 2022). NOX-2, an important NADPH oxidase isoform, catalyzes electron transfer to oxygen, generating a superoxide anion (Vermot et al., 2021). Excess superoxide reacts with NO, producing peroxynitrite, a highly toxic reactive nitrogen species (Pacher et al., 2007). Increased expression of NOX-2 has been reported in animal models with OAB (Alexandre et al., 2016; Akakpo et al., 2017; Alexandre et al., 2018; de Oliveira et al., 2022). Our study found increased NOX-2 expression in the bladder of PHZ-treated mice and elevated markers of oxidative and nitrosative stress, 4-HNE, and 3-NT. These results fit with our previous findings that demonstrate that PHZ-treated mice exhibit increased oxidative markers like 3-NT, 4-HNE, and NOX-2 in the penis (Iacopucci et al., 2022). Prior research has shown NOX-2 downregulation through NO-cGMP-dependent mechanisms (Teixeira et al., 2007). In contrast, NO inhibits NADPH oxidase-dependent superoxide anion production by a cGMP-independent mechanism without altering the protein expression of NOX-2 (Selemidis et al., 2007). In this context, this suggests that increased plasma hemoglobin may trigger oxidative stress elevation, reducing NO and cGMP bioavailability, as evidenced by reduced p-VASP (Ser-239).

In the present study, we used a PHZ-induced hemolysis model instead of transgenic mice with SCD for a few fundamental reasons. First, the PHZ-induced hemolysis model allows precise control over the onset and intensity of hemolysis, facilitating the direct correlation between hemolysis and the functional and molecular changes observed in the bladder. This precise control is important, as it allowed us to establish a direct relationship between intravascular hemolysis and the observed dysfunctions. Furthermore, this model is widely recognized for its ability to simulate key aspects of intravascular hemolysis observed in sickle cell disease, allowing specific investigation of the mechanisms underlying urinary complications. However, we recognize the value of transgenic models of SCD to study the disease in a broader context.

## 5 Conclusion

Our study is the first to show that intravascular hemolysis promotes voiding dysfunction correlated with alterations in the NO signaling pathway in the bladder, as evidenced by reduced levels of p-eNOS (Ser-1177), nNOS (Ser-1417), and p-VASP (Ser-239). The study also showed that intravascular hemolysis increases oxidative stress in the bladder. Our study indicates that intravascular hemolysis promotes OAB phenotypes similar to those observed in patients and mice with SCD, suggesting a potential mechanistic link. These findings suggest that pharmacologic interventions targeting intravascular hemolysis may ameliorate voiding dysfunction in SCD.

## Data Availability

The raw data supporting the conclusion of this article will be made available by the authors, without undue reservation.
